# Extracellular Matrix Dynamics in Hepatocarcinogenesis: a Comparative Proteomics Study of *PDGFC* Transgenic and *Pten* Null Mouse Models

**DOI:** 10.1371/journal.pgen.1002147

**Published:** 2011-06-23

**Authors:** Keane K. Y. Lai, Sufen Shang, Neha Lohia, Garrett C. Booth, Derek J. Masse, Nelson Fausto, Jean S. Campbell, Laura Beretta

**Affiliations:** 1 Molecular Diagnostics Program, Public Health Sciences Division, Fred Hutchinson Cancer Research Center, Seattle, Washington, United States of America; 2 Department of Pathology, University of Washington, Seattle, Washington, United States of America; The University of North Carolina at Chapel Hill, United States of America

## Abstract

We are reporting qualitative and quantitative changes of the extracellular matrix (ECM) and associated receptor proteomes, occurring during the transition from liver fibrosis and steatohepatitis to hepatocellular carcinoma (HCC). We compared two mouse models relevant to human HCC: *PDGFC* transgenic (Tg) and *Pten* null mice, models of disease progression from fibrosis and steatohepatitis to HCC. Using mass spectrometry, we identified in the liver of both models proteins for 26 collagen-encoding genes, providing the first evidence of expression at the protein level for 16 collagens. We also identified post-transcriptional protein variants for six collagens and lysine hydroxylation modifications for 14 collagens. Tumor-associated collagen proteomes were similar in both models with increased expression of collagens type IV, VI, VII, X, XIV, XV, XVI, and XVIII. Splice variants for *Col4a2*, *Col6a2*, *Col6a3* were co-upregulated while only the short form of *Col18a1* increased in the tumors. We also identified tumor specific increases of nidogen 1, decorin, perlecan, and of six laminin subunits. The changes in these non-collagenous ECM proteins were similar in both models with the exception of laminin β3, detected specifically in the *Pten* null tumors. *Pdgfa* and *Pdgfc* mRNA expression was increased in the *Pten* null liver, a possible mechanism for the similarity in ECM composition observed in the tumors of both models. In contrast and besides the strong up-regulation of integrin α5 protein observed in the liver tumors of both models, the expression of the six other integrins identified was specific to each model, with integrins α2b, α3, α6, and β1 up-regulated in *Pten* null tumors and integrins α8 and β5 up-regulated in the *PDGFC* Tg tumors. In conclusion, HCC–associated ECM proteins and ECM–integrin networks, common or specific to HCC subtypes, were identified, providing a unique foundation to using ECM composition for HCC classification, diagnosis, prevention, or treatment.

## Author Summary

The microenvironment can have a profound influence on cellular behavior and survival and on growth of developing tumor cells. We present the first comprehensive analysis of the extracellular matrix (ECM) and associated receptor proteomes, applied here to the study of hepatocellular carcinoma (HCC). This study demonstrates the utility of mass spectrometry-based approaches to characterize, at the protein level, gene families with extensive sequence homology, post-transcriptional regulations, and post-translational regulations. This is also the first study to analyze and compare liver proteome changes occurring during the transition from fibrosis and steatohepatitis, common preneoplastic conditions in humans, to HCC, using two mouse models. This approach identifies ECM and integrin components, which could play an important role in the early steps of hepatocarcinogenesis, and provides a path to identifying ECM–tumor cell networks that may contribute to the heterogeneous features of HCC.

## Introduction

Cirrhosis, the result of end-stage fibrosis, and steatohepatitis are common pre-neoplastic conditions associated with hepatocarcinogenesis [1]. It is therefore important to understand the mechanisms leading to the transition from fibrosis and steatosis to HCC. Mice with liver-specific transgenic (Tg) expression of platelet-derived growth factor-C (*PDGFC*) represent a relevant model for such a study as members of the PDGF family play major roles in angiogenesis and fibrosis [2], [3]. Moreover, these mice develop liver fibrosis resembling human alcoholic and nonalcoholic fatty liver disease, which precedes development of HCC [4], [5]. Another relevant model is mice with liver specific deletion of the phosphoinositide 3-kinase (PI3K)/phosphatase and tensin homolog (*Pten*). PTEN loss of function in hepatocytes leads to steatohepatitis, fibrosis and HCC later in life [6], [7]. While the liver tumors in *PDGFC* Tg mice show characteristics of HCC, the tumors in the *Pten* null model present a mixed phenotype of HCC and cholangiocarcinoma [8], [9]. Up to 40% of human HCCs potentially arise from progenitor-like tumor initiating cells and tend to have a more aggressive phenotype [10]. In addition, the presence of intermediate cells co-expressing both hepatocyte and biliary markers is associated with HCC occurrence [11] and acquisition of cholangiocarcinoma-like expression traits plays a critical role in the heterogeneous progression of HCC [12]. It is therefore of particular relevance to compare liver proteome changes in both the *PDGFC* Tg and the *Pten* null models.

Through mass-spectrometry-based profiling of the liver tissues collected at different disease stages in these two mouse models, we have characterized changes in the liver proteome occurring in fibrotic and steatotic tissue, as well as in tumors. We previously reported that the extensive mass-spectrometry-based approach we used in this study reaches depth and allows for quantitative estimates of protein abundance [13]. Changes in specific protein families or networks can be characterized as shown here for proteins of the extracellular matrix (ECM) and their receptors. The ECM is a key component of the microenvironment that is in immediate contact with the tumor cells and is a critical source for growth, survival, motility and angiogenic factors that significantly affect tumor biology and progression. In addition, cell adhesion to the ECM through integrins and other cell surface receptors triggers intracellular signaling pathways that can regulate cell cycle progression, migration and differentiation. While hepatic ECM has been extensively studied in the context of liver fibrosis, little attention has been given to the role of the ECM in the early steps of hepatocarcinogenesis. Therefore, delineating and comparing the liver proteome changes of ECM components in two mouse models of liver cancer represents a unique and important contribution to our understanding of the molecular mechanisms of early hepatocarcinogenesis, and to ongoing efforts to identify novel diagnostic and therapeutic targets.

## Results

### Tumor Development in the *PDGFC* Tg and *Pten* Null Mice and Liver Proteome Profiling

**Figure 1:**
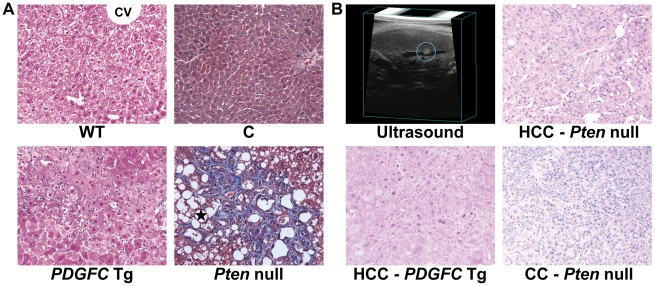
Collagen deposition and tumor morphology in *PDGFC* Tg and *Pten* null liver. A) Masson's trichrome stained liver sections showing hepatic pericellular collagen deposition in 6-month-old *PDGFC* Tg and *Pten* null mice compared to WT littermates and control mice. Black star: lipid droplets in *Pten* null livers; CV: central vein. B) H&E stained liver sections showing HCC formation in a 8-month-old *PDGFC* Tg mouse detected by ultrasound, and HCC and CC formation in a 9-month-old *Pten* null mouse. Magnification: ×200.

The liver specific *PDGFC* Tg and *Pten* null mouse models reproduce the steps of HCC development observed in humans progressing from steatohepatitis and fibrosis to hepatocyte dysplasia and tumorigenesis. These events are associated with significant modification of the stroma and associated extracellular matrix. Preceding tumor development, there is accumulation of collagen fibers in the liver of these mice (Figure 1A). Steatosis is also particularly pronounced in the *Pten* null liver as shown by the accumulation of lipid droplets (Figure 1A). Interestingly, the phenotypes of the tumors are different in these two models with characteristics of HCC in the *PDGFC* Tg model and with mixed cell characteristics of HCC and cholangiocarcinoma in the *Pten* null mice (Figure 1B). Extensive mass spectrometry analysis following a multi-dimensional protein separation strategy composed of two-dimensional HPLC followed by SDS-PAGE was applied as described [13] to livers collected from these two models at the fibrosis and steatosis stage as well as on small HCCs. For each sample group, livers from three or four mice were pooled. A total of 10,707 protein isoforms, products of 8,278 individual genes were identified with high confidence. For each identified protein, protein abundance was calculated using the frequency of tandem mass spectra assigned to that protein. We previously reported that this label-free approach provides a good estimate of protein abundance in liver [13].

### Collagen Protein Identification and Quantification

**Figure 2:**
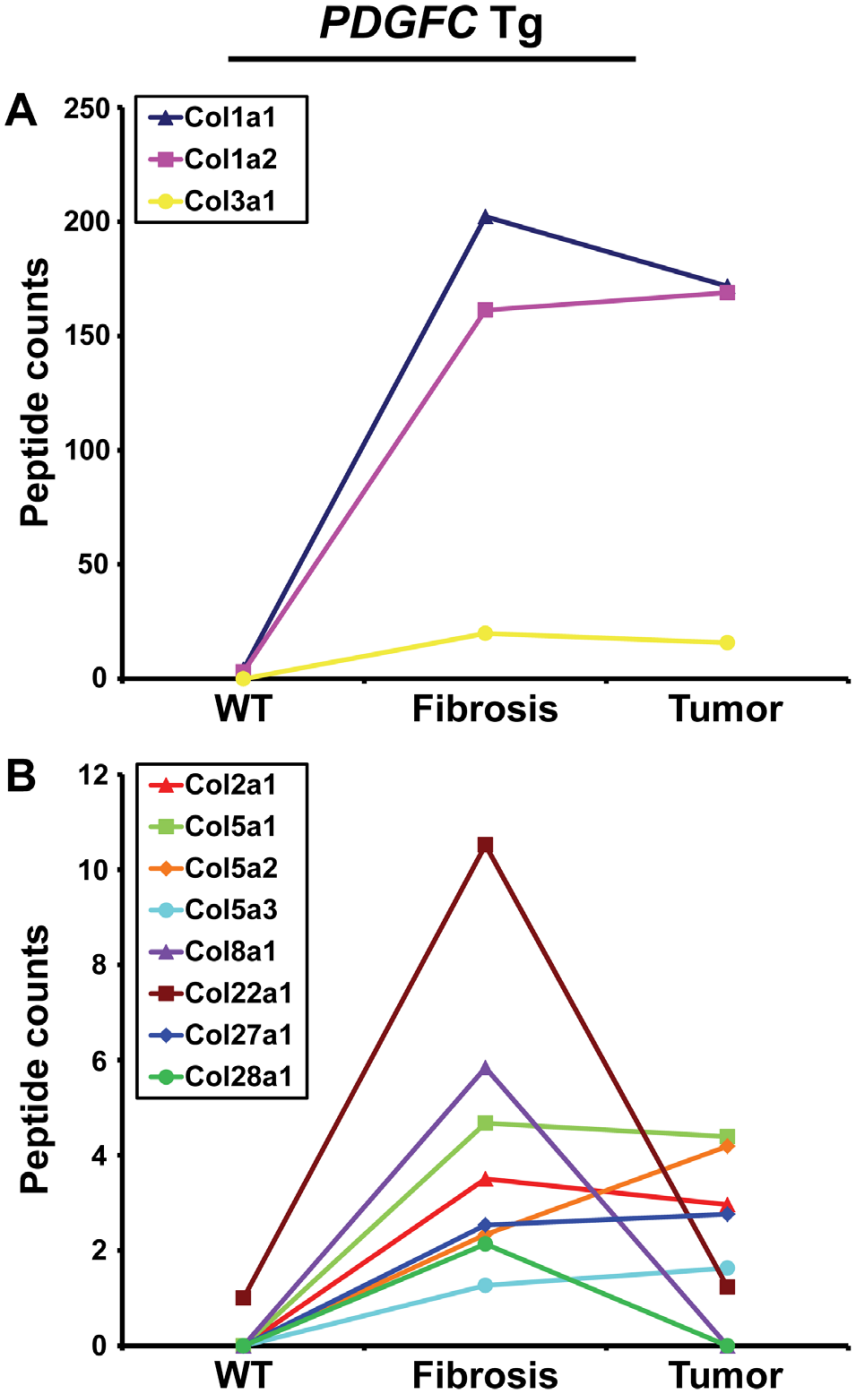
Collagen proteins up-regulated in *PDGFC* Tg fibrotic liver. The figure includes the collagens identified as up-regulated in *PDGFC* Tg fibrotic liver compared to *PDGFC* Tg tumors and WT liver. For each protein, the abundance is shown as the total number of tandem mass spectra assigned to that protein. The collagens of high abundance are shown in panel A and those of lower abundance are shown in panel B.

Out of the 44 alpha chains of the murine collagen family, a total of 26, corresponding to 16 collagen types, were identified: COL1A1, COL1A2, COL2A1, COL3A1, COL4A1, COL4A2, COL4A3, COL4A4, COL4A5, COL4A6, COL5A1, COL5A2, COL5A3, COL6A1, COL6A2, COL6A3, COL7A1, COL8A1, COL10A1, COL14A1, COL15A1, COL16A1, COL18A1, COL22A1, COL27A1, COL28A1 (Table 1). All 26 collagens were identified with ProteinProphet scores of 0.97 or higher corresponding to a false discovery rate of 0.003 and all 26 collagens were identified in both mouse models. For 16 of these 26 collagens, this study represents to date the first evidence at the protein level (www.uniprot.org). The information on protein annotation, peptide numbers and sequences is summarized in Table S1. As expected, collagens types I and III were predominant in abundance. Among the remaining collagens, collagens types IV, V and VI were the most abundant. Significant changes in abundance during disease progression were observed for all 26 collagen proteins. In human liver, type I and III collagen levels increase up to eight times during fibrogenesis, with a significantly higher increase of type I collagen than of type III collagen. Similarly, type I and III collagen levels strongly increased in the fibrotic liver of 2-month-old *PDGFC* Tg mice with a significantly higher increase of COL1A1 and COL1A2 than of COL3A1 (Figure 2A). Eight collagens of lower abundance were also up-regulated in the *PDGFC* Tg fibrotic liver. These included: COL2A1, COL5A1, COL5A2, COL5A3, COL8A1, COL22A1, COL27A1 and COL28A1 (Figure 2B).

**Table 1:** Collagen proteins identified in *PDGFC* Tg and *Pten* null liver.

Protein names	Gene names	# Unique peptides
Collagen alpha-1(I) chain	*Col1a1*	29
Collagen alpha-2(I) chain	*Col1a2* *	30
Collagen alpha-1(II) chain	*Col2a1*	8
Collagen alpha-1(III) chain	*Col3a1* *	11
Collagen alpha-1(IV) chain	*Col4a1* *	11
Collagen alpha-2(IV) chain	*Col4a2* *	11
Collagen alpha-3(IV) chain	*Col4a3*	4
Collagen alpha-4(IV) chain	*Col4a4* *	7
Collagen alpha-5(IV) chain	*Col4a5* *	3
Collagen alpha-6(IV) chain	*Col4a6* *	3
Collagen alpha-1(V) chain	*Col5a1* *	10
Collagen alpha-2(V) chain	*Col5a2*	5
Collagen alpha-3(V) chain	*Col5a3* *	2
Collagen alpha-1(VI) chain	*Col6a1* *	17
Collagen alpha-2(VI) chain	*Col6a2* *	24
Collagen alpha-3(VI) chain	*Col6a3*	67
Collagen alpha-1(VII) chain	*Col7a1* *	4
Collagen alpha-1(VIII) chain	*Col8a1*	1
Collagen alpha-1(X) chain	*Col10a1* *	2
Collagen alpha-1(XIV) chain	*Col14a1* *	25
Collagen alpha-1(XV) chain	*Col15a1*	7
Collagen alpha-1(XVI) chain	*Col16a1* *	6
Collagen alpha-1(XVIII) chain	*Col18a1*	14
Putative protein COL22A1	*Col22a1* *	5
Collagen alpha-1(XXVII) chain	*Col27a1*	6
Collagen alpha-1(XXVIII) chain	*Col28a1*	5

*No previous evidence at the protein level reported in UniProtKB (www.uniprot.org).

### Collagen Protein Abundance Changes in Tumors

**Figure 3:**
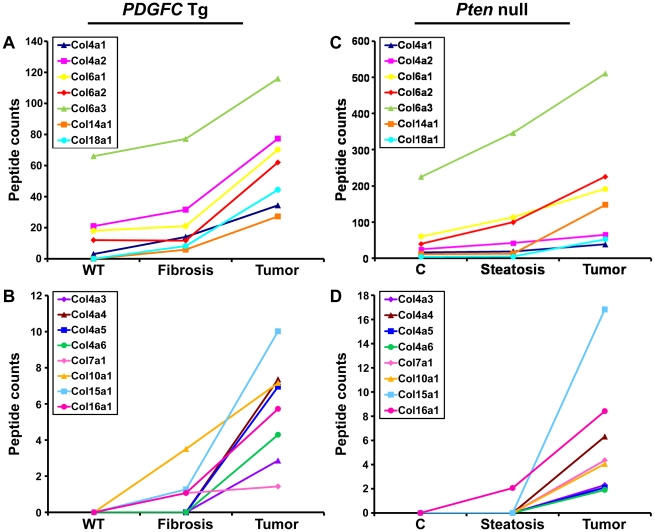
Collagen proteins up-regulated in *PDGFC* Tg and *Pten* null tumors. A,B) The figure includes the collagens identified as up-regulated in *PDGFC* Tg tumors compared to *PDGFC* Tg fibrotic and WT liver. C,D) The figure includes the collagens identified as up-regulated in *Pten* null tumors compared to *Pten* null steatotic and control liver. For each protein, the abundance is shown as the total number of tandem mass spectra assigned to that protein. The collagens of high abundance are shown in panels A and C and those of lower abundance are shown in panels B and D.

**Figure 4:**
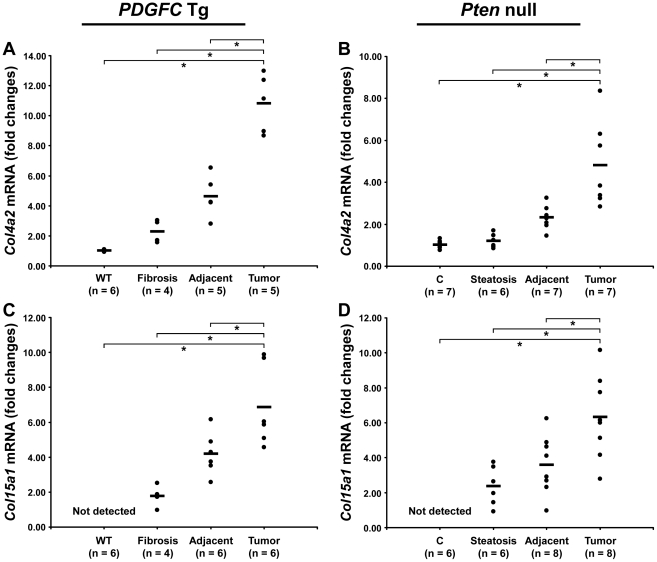
Up-regulation of *Col4a2* and *Col15a1* mRNAs in *PDGFC* Tg and *Pten* null tumors. A,C) Expression of *Col4a2* and *Col15a1* mRNAs was measured by quantitative PCR in *PDGFC* Tg fibrotic liver, in *PDGFC* Tg tumor and adjacent tissue, and in age-matched WT liver. B,D) Similarly, expression of *Col4a2* and *Col15a1* mRNAs was measured in *Pten* null steatotic liver, in *Pten* null tumor and adjacent tissue, and in age-matched control liver. Expression in the disease groups is represented as fold changes over the mean of expression in the control groups.

The protein abundance of the remaining 15 identified collagens increased in the tumors of 8-month old *PDGFC* Tg mice as shown in Figure 3A for the proteins of moderate to high abundance (COL4A1, COL4A2, COL6A1, COL6A2, COL6A3, COL14A1 and COL18A1) and in Figure 3B for the proteins of lower abundance (COL4A3, COL4A4, COL4A5, COL4A6, COL7A1, COL10A1, COL15A1 and COL16A1). The same abundance changes were observed for these 15 collagens in the tumors of 9-month old *Pten* null mice (Figure 3C, 3D). In both models, the tumor-associated abundance increase was particularly significant for collagens type IV, VI, XIV, XV and XVI. Validation at the transcript level was performed for *Col4a2* and *Col15a1*. *Col4a2* mRNA was strongly up-regulated in tumors of both models with 10.8-fold increase (p = 0.008) in *PDGFC* Tg mice (Figure 4A) and 4.8-fold increase (p = 0.002) in *Pten* null mice (Figure 4B). *Col4a2* mRNA expression was also significantly higher in tumors compared to adjacent tissue of both models (p = 0.01 in *PDGFC* Tg mice and p = 0.001 in *Pten* null mice). *Col15a1* mRNA was not detected in control liver tissues and was only weakly expressed in fibrotic and steatotic liver in both models. Its expression was significantly increased in tumors in both models with 6.8-fold increase (p = 0.009) in *PDGFC* Tg mice (Figure 4C) and 6.3-fold increase (p = 0.003) in *Pten* null mice (Figure 4D). *Col15a1* mRNA expression was also significantly higher in tumors compared to adjacent tissue in both models (p = 0.04 in *PDGFC* Tg mice and p = 0.02 in *Pten* null mice).

### Collagen Post-Transcriptional Variants

**Figure 5:**
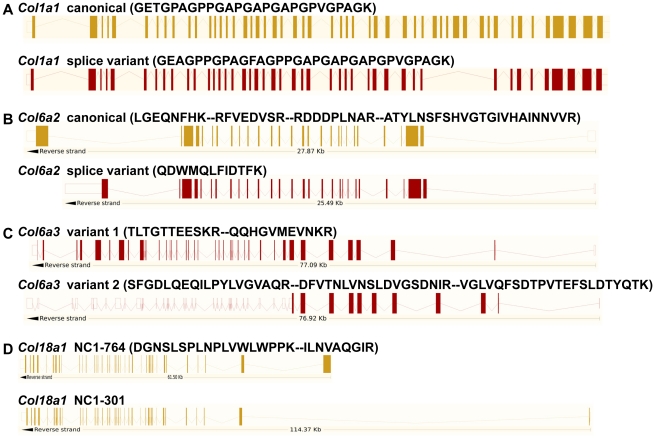
Identification of peptides specific to post-transcriptional variants for *Col1a1*, *Col6a2*, *Col6a3*, and *Col18a1*. The figure shows the exon structures of post-transcriptional variants for *Col1a1*, *Col6a2*, *Col6a3* and *Col18a1* and the associated specific peptides identified by mass spectrometry in the *PDGFC* Tg and *Pten* null liver.

**Figure 6:**
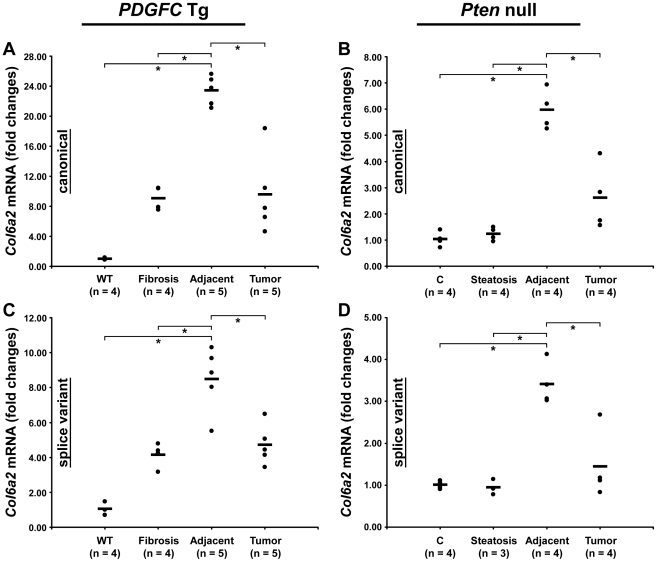
Expression of *Col6a2* mRNA variants upon disease progression in *PDGFC* Tg and *Pten* null liver. A,C) Expression of *Col6a2* mRNA splice variants was measured by quantitative PCR in *PDGFC* Tg fibrotic liver, in *PDGFC* Tg tumor and adjacent tissue and in age-matched WT liver. B,D) Similarly, expression of *Col6a2* mRNA splice variants was measured in *Pten* null steatotic liver, in *Pten* null tumor and adjacent tissue and in age-matched control liver. Expression in the disease groups is represented as fold changes over the mean of expression in the control groups.

**Figure 7:**
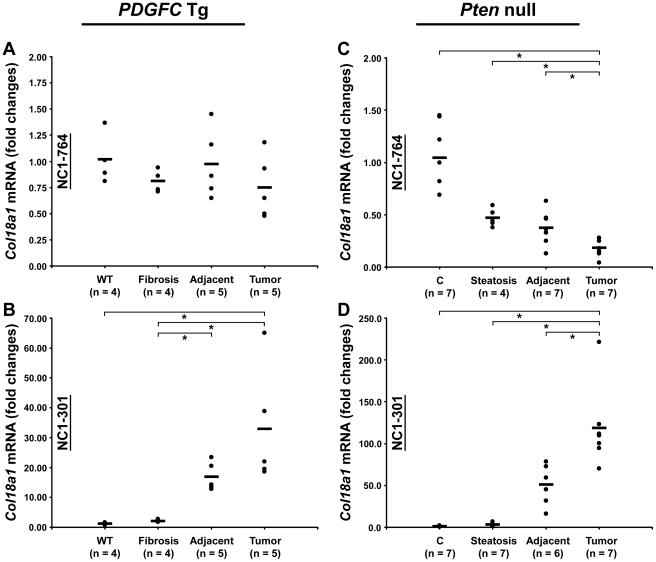
Expression of *Col18a1* mRNA variants upon disease progression in *PDGFC* Tg and *Pten* null liver. A,C) Expression of *Col18a1* mRNA variants NC1-764 and NC1-301 was measured by quantitative PCR in *PDGFC* Tg fibrotic liver, in *PDGFC* Tg tumor and adjacent tissue and in age-matched WT liver. B,D) Similarly, expression of *Col18a1* mRNA variants NC1-764 and NC1-301 was measured in *Pten* null steatotic liver, in *Pten* null tumor and adjacent tissue and in age-matched control liver. Expression in the disease groups is represented as fold changes over the mean of expression in the control groups.

Peptides specific to post-transcriptional variants were identified for *Col1a1*, *Col6a2*, *Col6a3* and *Col18a1* (Figure 5). These variants result from alternative splicing for *Col1a1*, *Col6a2* and *Col6a3* and from alternative promoter usage for *Col18a1*. Validation at the transcript level was performed for *Col6a2* and *Col18a1* using primers specific to the post-transcriptional variants. *Col6a2* canonical mRNA was strongly up-regulated in tissue adjacent to tumors in both models with 23.4-fold increase (p = 0.01) in *PDGFC* Tg mice (Figure 6A) and 6.0-fold increase (p = 0.02) in *Pten* null mice (Figure 6B). A correlated up-regulation was observed for *Col6a2* splice variant with 8.5-fold increase (p = 0.01) in *PDGFC* Tg mice (Figure 6C) and 3.4-fold increase (p = 0.02) in *Pten* null mice (Figure 6D). *Col18a1* canonical mRNA also called NC1-764, was unchanged in liver tissue and tumors of *PDGFC* Tg mice (Figure 7A) and decreased in tumors of *Pten* null mice (5.5-fold, p = 0.002) (Figure 7B). In contrast, *Col18a1* variant NC1-301 strongly increased in tumors in both models with 32.8-fold increase (p = 0.01) in *PDGFC* Tg mice (Figure 7C) and 118.7-fold increase (p = 0.0006) in *Pten* null mice (Figure 7D). *Col18a1* variant NC1-301 also strongly increased in adjacent tissue in both models with 16.9-fold increase (p = 0.01) in *PDGFC* Tg mice (Figure 7C) and 50.7-fold increase (p = 0.001) in *Pten* null mice (Figure 7D).

### Lysine Hydroxylation of Collagens

**Figure 8:**
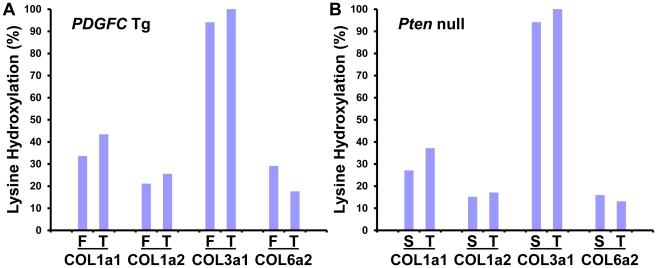
Percentage of peptides with lysine hydroxylation identified for COL1A1, COL1A2, COL3A1, and COL6A2. In A) *PDGFC* Tg fibrotic (F) and tumors (T) and in B) *Pten* null steatotic liver (S) and tumors (T).

Lysine hydroxylation is a well-known post-translational modification of type I, III and V collagens and contributes to matrix remodeling and stiffening. We investigated whether lysine hydroxylation occurs on other collagens and changes in abundance during tumor development, by researching the mass spectrometry raw data using criteria allowing for the identification of lysine hydroxylation modifications. Extensive lysine hydroxylation modification was observed as expected for COL1A1, COL1A2, COL3A1 and COL5A1 with 9, 12, 7 and 5 modified lysine residues identified, respectively. Other collagens with modified lysine residues included all six type IV collagens, COL6A2, COL16A1 and COL27A1 (Table 2). The lysine hydroxylation status was particularly high for COL3A1 with 94% of identified peptides presenting with lysine modifications in both *PDGFC* Tg fibrotic liver and *Pten* null steatotic liver; and with 100% of identified peptides presenting with lysine modifications in the tumors collected from both models (Figure 8). The lysyl hydroxylation status of COL1A1 and COL1A2 also slightly increased in the tumors compared to the fibrotic and steatotic livers in both models increasing from 33% to 43% for COL1A1 and from 21% to 25% for COL1A2 in *PDGFC* Tg mice and increasing from 27% to 37% for COL1A1 and from 15% to 17% for COL1A2 in *Pten* null mice (Figure 8). Inversely, the lysyl hydroxylation status of COL6A2 slightly decreased in the tumors compared to the fibrotic and steatotic livers in both models decreasing from 29% to 17% in *PDGFC* Tg mice and from 15% to 13% in *Pten* null mice (Figure 8). For the type IV collagens, the hydroxylation status was below 5%. Low hydroxylation was also found for COL5A1, COL5A2, COL16A1 and COL27A1.

**Table 2:** Collagens with lysine hydroxylation modifications identified in *PDGFC* Tg and *Pten* null liver.

Protein names	Gene names	# sites	Lysine residues
Collagen alpha-1(I) chain	*Col1a1*	9	K(160), K(266), K(386), K(419), K(437), K(457), K(541), K(575), K(1085)
Collagen alpha-2(I) chain	*Col1a2*	12	K(360), K(366), K(386), K(504), K(512), K(744), K(747), K(750), K(753), K(852), K(1014), K(1020)
Collagen alpha-1(III) chain	*Col3a1*	7	K(502), K(742), K(756), K(823), K(859)*, K(1030), K(1149)
Collagen alpha-1(IV) chain	*Col4a1*	1	K(1651)
Collagen alpha-2(IV) chain	*Col4a2*	2	K(165), K(932)
Collagen alpha-3(IV) chain	*Col4a3*	1	K(90)
Collagen alpha-4(IV) chain	*Col4a4*	1	K(1400)
Collagen alpha-5(IV) chain	*Col4a5*	2	K(664), K(1008)
Collagen alpha-6(IV) chain	*Col4a6*	1	K(790)
Collagen alpha-1(V) chain	*Col5a1*	5	K(535), K(905), K(963), K(1433), K(1792)
Collagen alpha-2(V) chain	*Col5a2*	1	K(584)
Collagen alpha-2(VI) chain	*Col6a2*	2	K(408), K(500)
Collagen alpha-1(XVI) chain	*Col16a1*	2	K(496), K(683)
Collagen alpha-1(XXVII) chain	*Col27a1*	2	K(202), K(1477)

### Non-Collagenous ECM Components

**Figure 9:**
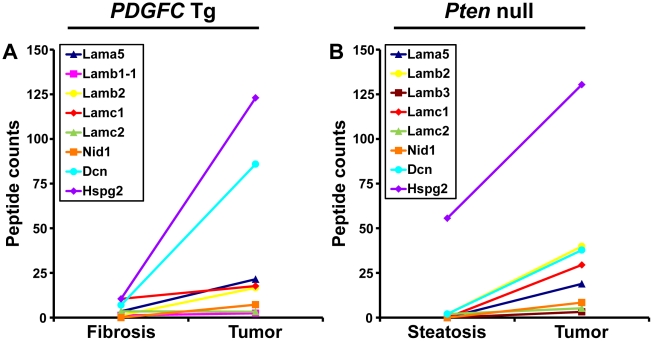
Non-collagenous ECM proteins up-regulated in *PDGFC* Tg and *Pten* null tumors. A) The figure includes the non-collagenous ECM proteins identified as up-regulated in *PDGFC* Tg tumors compared to *PDGFC* Tg fibrotic liver. B) The figure includes the non-collagenous ECM proteins identified as up-regulated in *Pten* null tumors compared to *Pten* null steatotic liver. For each protein, the abundance is shown as the total number of tandem mass spectra assigned to that protein.

**Figure 10:**
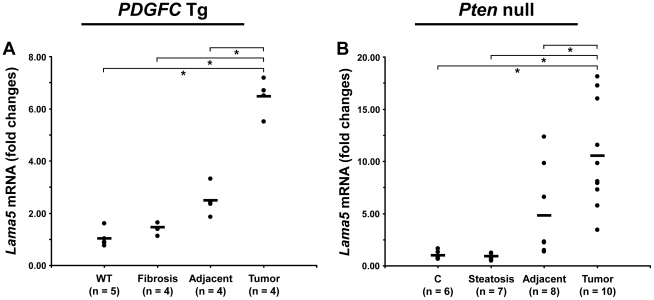
Up-regulation of laminin α5 mRNA in *PDGFC* Tg and *Pten* null tumors. A) Expression of laminin α5 (*Lama5*) mRNA was measured by quantitative PCR in *PDGFC* Tg fibrotic liver, in *PDGFC* Tg tumor and adjacent tissue, and in age-matched WT liver. B) Similarly, expression of laminin α5 (*Lama5*) mRNA was measured in *Pten* null steatotic liver, in *Pten* null tumor and adjacent tissue, and in age-matched control liver. Expression in the disease groups is represented as fold changes over the mean of expression in the control groups.

**Figure 11:**
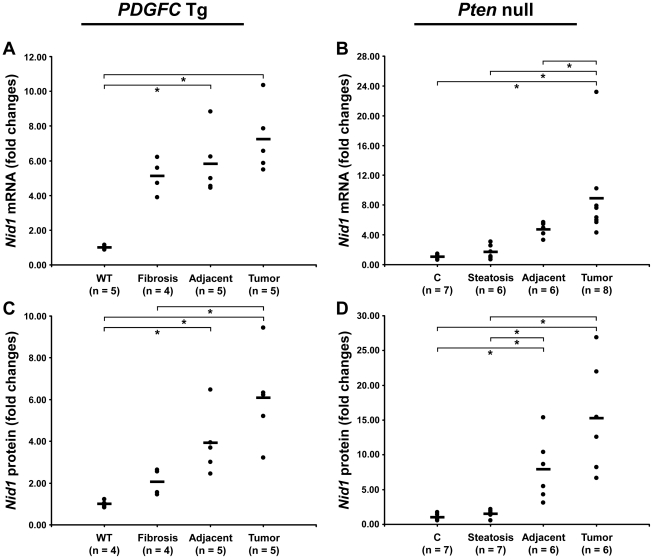
Up-regulation of nidogen 1 mRNA and protein in *PDGFC* Tg and *Pten* null tumors. A,C) Expression of nidogen 1 mRNA and protein was measured by quantitative PCR and Western-blot, in *PDGFC* Tg fibrotic liver, in *PDGFC* Tg tumor and adjacent tissue and in age-matched WT liver. B,D) Similarly, expression of nidogen 1 mRNA and protein was measured in *Pten* null steatotic liver, in *Pten* null tumor and adjacent tissue, and in age-matched control liver. Expression in the disease groups is represented as fold changes over the mean of expression in the control groups.

The ECM is also composed of non-collagenous proteins such as laminins. Laminins are large macromolecules constituted by the association of one α, one β and one γ chain. Laminin α5, laminin β2 and laminin γ1 were up-regulated in the tumors of both mouse models (Figure 9), suggesting that laminin 521 (previously called laminin 11) is the most abundant laminin in HCC. Laminin β3 and laminin γ2 were specifically up-regulated in *Pten* null tumors while laminin β1 was specifically up-regulated in *PDGFC* Tg tumors (Figure 9). Perlecan, also known as HSPG2, decorin and nidogen 1 were up-regulated in tumors of both models. Validation at the transcript level was performed for laminin α5 and nidogen 1. Laminin α5 mRNA was only weakly expressed in fibrotic and steatotic liver in both models but was significantly up-regulated in tumors in both models (6.4-fold (p = 0.01) in *PDGFC* Tg mice and 10.5-fold (p = 0.0002) in *Pten* null mice) (Figure 10). Laminin α5 mRNA expression was also significantly higher in tumors compared to adjacent tissues, in both models (p = 0.02 in *PDGFC* Tg mice and p = 0.03 in *Pten* null mice) (Figure 10). Nidogen 1 mRNA was increased by 7.2-fold (p = 0.008) and by 8.9-fold (p = 0.0003) in tumors from *PDGFC* Tg and *Pten* null mice, respectively (Figure 11A, 11B). Similarly, nidogen 1 protein was increased by 6.1-fold (p = 0.01) and by 15.3-fold (p = 0.001) in tumors from *PDGFC* Tg and *Pten* null mice, respectively (Figure 11C, 11D).

### PDGF Expression in *Pten* Null Mice

**Figure 12:**
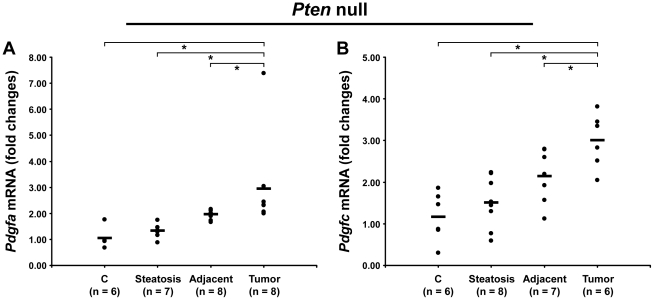
Up-regulation of *Pdgfa* and *Pdgfc* mRNAs in *Pten* null tumors. Expression of A) *Pdgfa* mRNA and B) *Pdgfc* mRNA was measured in *Pten* null steatotic liver, in *Pten* null tumor and adjacent tissue, and in age-matched control liver. Expression in the disease groups is represented as fold changes over the mean of expression in the control groups.

Because of the similarity in ECM composition in both models, we investigated whether PDGF ligands were up-regulated in *Pten* null liver. While *Pdgfb* mRNA was undetected, both *Pdgfa* and *Pdgfc* mRNAs were up-regulated in *Pten* null tumors by 3.0-fold (p = 0.0007 and p = 0.002, respectively) and in adjacent tissue by 2-fold (p = 0.003 and p = 0.02, respectively) (Figure 12). The up-regulation of both PDGFA and PDGFC may therefore explain the common ECM changes observed in the *PDGFC* Tg and *Pten* null tumors and adjacent tissue.

### ECM Receptors

**Figure 13:**
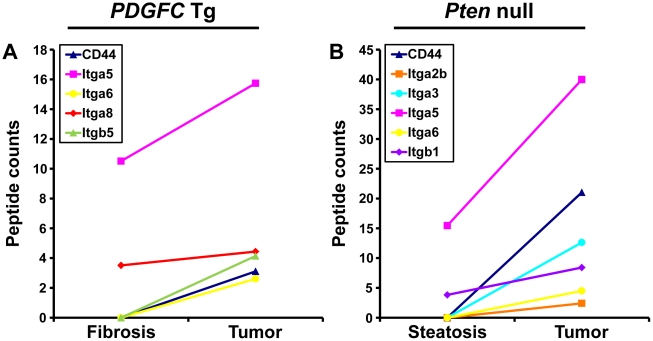
CD44 and integrin proteins up-regulated in *PDGFC* Tg and *Pten* null tumors. A) The figure includes CD44 and the integrin subunits identified up-regulated in *PDGFC* Tg tumors compared to *PDGFC* Tg fibrotic liver. B) The figure includes CD44 and the integrin subunits identified as up-regulated in *Pten* null tumors compared to *Pten* null steatotic liver. For each protein, the abundance is shown as the total number of tandem mass spectra assigned to that protein.

**Figure 14:**
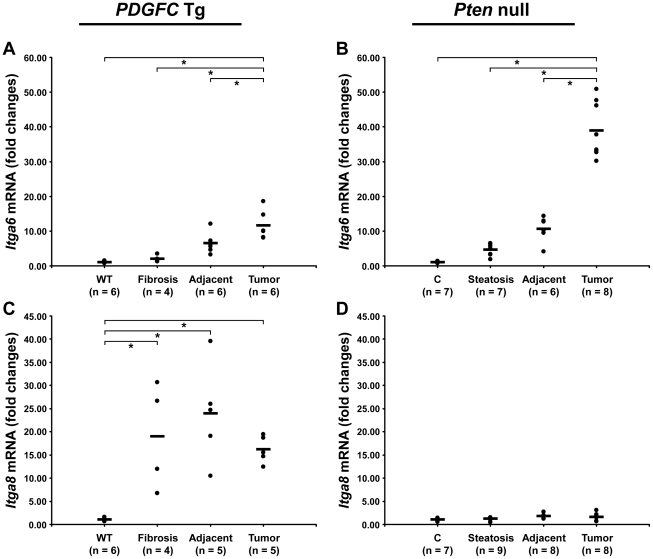
Expression of integrin α6 and α8 mRNAs upon disease progression in *PDGFC* Tg and *Pten* null liver. A,C) Expression of *Itga6* and *Itga8* mRNAs was measured by quantitative PCR in *PDGFC* Tg fibrotic liver, in *PDGFC* Tg tumor and adjacent tissue, and in age-matched WT liver. B,D) Similarly, expression of *Itga6* and *Itga8* mRNAs was measured in *Pten* null steatotic liver, in *Pten* null tumor and adjacent tissue, and in age-matched control liver. Expression in the disease groups is represented as fold changes over the mean of expression in the control groups.

Cell-ECM interactions are largely mediated through receptors called integrins made up of α and β chains. While integrin α5 was the most abundant and commonly up-regulated integrin chain in the tumors of both mouse models, the pattern of the other identified integrins was significantly different between the two models (Figure 13). Integrins α2b, α3 and β1 were specific to *Pten* null tumors while integrins α8 and β5 were specific to *PDGFC* Tg tumors. The up-regulation of integrin α6 and of CD44 was also much stronger in *Pten* null tumors compared to *PDGFC* Tg tumors. The differential expression of integrins α6 and α8 was further validated. Integrin α6 mRNA was increased by 39.0-fold (p = 0.001) in *Pten* null tumors and by 11.6-fold (p = 0.002) in *PDGFC* Tg tumors (Figure 14A, 14B). Integrin α8 mRNA was increased in *PDGFC* Tg liver tissue at all disease stages by 16.2- to 24.0-fold (Figure 14C) but remained unchanged in *Pten* null liver (Figure 14D).

**Figure 15:**
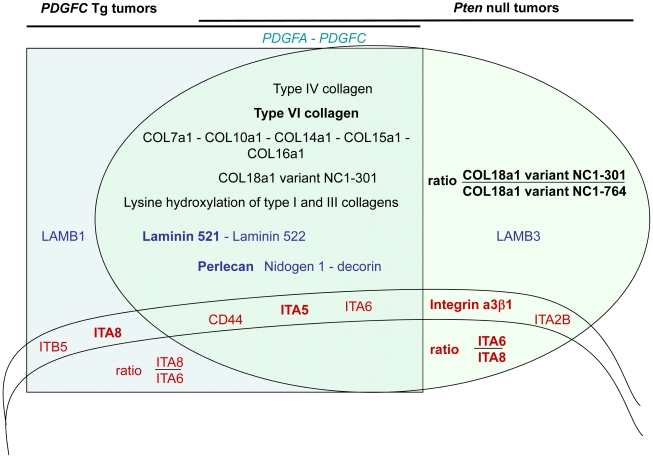
Schema summarizing the ECM protein components and their receptors identified as up-regulated in *PDGFC* Tg and *Pten* null tumors. All ECM proteins and associated receptors identified as up-regulated in the mice tumors are shown as follows: in black for collagens, in blue for non-collagenous ECM proteins and in red for ECM receptors. Their position in the graph indicates whether they were commonly identified in the tumors of both mouse models (overlapping section between the light green oval representing the *Pten* null tumors and the light blue square representing the *PDGFC* Tg tumors) or identified specifically in one tumor type (light green section for *Pten* null tumors and light blue section for *PDGFC* Tg tumors). These two latter non-overlapping sections of the graph also indicate ratios of selected proteins that may have utility in discriminating between the *PDGFC* Tg and the *Pten* null tumors. The proteins in bold are those identified with higher abundance.

In summary (Figure 15), this study identified collagens type IV, VI, VII, X, XIV, XV, XVI and the short variant of COL18A1, NC1-301, as tumor-associated collagens in HCC. Laminin 521 was the most abundant laminin in HCC and integrin α5 the most abundant integrin subunit. High ratios of COL18A1 variant NC1-301 over COL18A1 variant NC1-764, high ratios of integrin α6 over integrin α8 and high levels of integrin α3 were specifically observed in the *Pten* null tumors.

## Discussion

The microenvironment can have profound influences on cellular behavior, survival and growth of developing tumor cells [14]. Increased rigidity of the ECM is commonly associated with HCC [15] and ECM deposition and matrix remodeling has been shown to affect liver progenitor cell expansion [16]. We characterized and compared ECM protein changes occurring during tumor development in the *PDGFC* Tg mouse model, a model of HCC associated with fibrosis and angiogenesis [4] and in the *Pten* null mouse model, a model of liver tumors of mixed cholangio- and hepatocytic features [6]–[8], [17]. This study represents the most comprehensive analysis of the ECM and associated receptor proteome reported to date, and demonstrates the utility of mass spectrometry-based approaches to study gene families with extensive sequence homology, post-transcriptional and post-translational regulations. It is also the first study to analyze and compare proteome changes occurring during the transition from fibrosis and steatosis to HCC in two mouse models.

Collagens, the most abundant structural components of the ECM are homo- and heterotrimeric molecules whose subunits, the alpha chains, are distinct gene products. Forty-four different alpha chains have been sequenced, several of them being differentially spliced, which adds to the diversity of the collagen family. To date, 28 different combinations of the alpha chains (collagen types I–XXVIII) have been identified or predicted to exist (www.uniprot.org). While only ten collagen types have been described in the liver [15], this extensive proteomic study resulted in the identification of 16 types. Fibril-forming types I and III collagens are predominantly synthesized by hepatic stellate cells and are used as markers for liver fibrogenesis. In the fibrotic liver of *PDGFC* Tg mice, type I and III collagen levels strongly increased with a significantly higher increase of type I collagen than of type III collagen changing the I/III ratio from 1∶1 in the healthy liver to over 2∶1, as observed in human fibrotic liver. Collagens type V (COL5A1, COL5A2 and COL5A3) and type II (COL2A1), the other fibril-forming collagens, were also up-regulated in the fibrotic liver of the *PDGFC* Tg mice. This ECM composition in the fibrotic liver of *PDGFC* Tg mice is consistent with a signature of activated hepatic stellate cells, a hallmark of *PDGFC* Tg mice [4]. Out of the 26 alpha chains identified, 15 were up-regulated in tumors of both models. These include the six alpha chains of collagen IV, the three alpha chains of collagen VI, COL7A1, COL10A1, COL14A1, COL15A1, COL16A1 and COL18A1. Collagen VI, a component of microfibrillar structures in many tissues, is a heterotrimer with the chain composition (6a1)(6a2)(6a3). Type VI collagen binds cells and may be involved in cell migration and differentiation and embryonic development. All collagen VI subunits, including splice variants for COL6A2 and COL6A3, were up-regulated in the tumors and adjacent tissue of both models. The most abundant structural component of basement membranes is collagen IV. The six different alpha chains 4a1–4a6 were up-regulated in the tumors of both mouse models. Besides the heterotrimeric molecule (4a1)2(4a2) composed of the two most abundant collagen IV subunits, the other combinations between alpha chains, particularly those including the subunits of minor abundance, are not yet established. Whereas COL4A1 and COL4A2 are found in all basement membranes studied, COL4A3, COL4A4 and COL4A5 are found only in a subset of basement membranes and are always found together [18]. Strong deposition of collagen type IV was described in dysplastic areas and small HCCs in human cirrhotic livers indicative of early events in hepatocarcinogenesis [19]. The multiplexin collagens XV and XVIII are also localized to basement membranes. COL15A1 was the collagen alpha chain that showed the stronger up-regulation in both the *Pten* null and *PDGFC* Tg tumors.

COL18A1 was also up-regulated in the mice tumors. Interestingly, increases in this protein correlated with the increase of a specific isoform of *Col18a1* mRNA, isoform NC1-301, resulting from alternative promoter usage. NC1-301 mRNA was increased by over 30-fold in *PDGFC* Tg tumors and over 100-fold in *Pten* null tumors. In contrast, the canonical *Col18a1* mRNA, NC1-764, was unchanged or slightly decreased in the *PDGFC* Tg tumors and strongly decreased in *Pten* null tumors. It was previously reported that NC1-764 mRNA expression decreases in advanced HCCs [20] and that cholangiocarcinoma cells expressed NC1-301 which was deposited in tumor basement membrane [21]. This is in good agreement with the changes we observed in both COL18A1 isoforms in the mice tumors, with a greater ratio NC1-301/NC1-764 in *Pten* null tumors compared to *PDGFC* Tg tumors. These results suggest that the ratio of COL18A1 isoforms could directly correlate with the expansion of intermediate cells co-expressing both hepatocytes and biliary markers. Finally, collagen VII, the main constituent of anchoring fibrils, was also up-regulated in tumors of both models. It has been reported that human epidermal cells devoid of collagen VII did not form tumors in mice, whereas those retaining the specific N-terminal NC1 domain were tumorigenic [22].

Other glycoproteins in basement membranes such as laminins and nidogen 1 increased in the mice tumors. Nidogens are believed to connect laminin and collagen IV networks, hence stabilizing the basement membrane structure and appear critical for anchoring other components such as perlecan. At present, five laminin α (α1–α5), three β (β1–β3) and three γ (γ1–γ3) chains and 16 trimers have been characterized in mouse and human [23]. Based on the chain identification, laminins 511 or 521 (previously called laminin 10/11) and laminin 522 are likely up-regulated in *PDGFC* Tg and *Pten* null tumors. It was reported that laminins containing the α5 chain serve as functional regulators of HCC progression [24]. Up-regulation of laminin β3, a major component of laminin 332 (previously called laminin 5) was observed specifically in *Pten* null tumors. Interestingly, laminin β3 was reported up-regulated in cholangiocarcinoma cell lines compared to HCC cell lines [25] and laminin 332 is present in almost all intrahepatic cholangiocarcinoma cases [26].

The global expression of the ECM in liver during tumor development results from the combined expression profiles of tumor cells, stromal cells, and non-tumor hepatocytes. Activated hepatic stellate cells and myofibroblasts express a wide spectrum of ECM molecules but an important fraction of ECM is also synthesized by other liver cells, notably sinusoidal and portal endothelia, bile duct epithelia and hepatocytes [27], [28]. While this study increases our knowledge of HCC-specific matrix composition, future studies should focus on the cellular distribution of the described proteins. Overall, beside a subset of laminins, the ECM changes were remarkably similar in the tumors and adjacent tissues of both mouse models, suggesting a common molecular and cellular mechanism. We therefore investigated the possibility that PDGF factors were up-regulated in *Pten* null mice. While *Pdgfb* mRNA was undetected in *Pten* null liver, both *Pdgfa* and *Pdgfc* transcripts were increased in *Pten* null tumors and adjacent tissues. We also observed an up-regulation of both PDGF receptor alpha and PDGF receptor beta in *Pten* null tumors and adjacent tissues (LB, personal communication).

Most cell types have the ability to bind to the surrounding ECM and certain ECM components can transmit signals to cells via transmembrane receptors [29]. Such matrix sensors are mainly integrins. Integrins comprise a large family of cell surface glycoproteins which consist of alpha and beta subunits and that regulate cell adhesion, migration, proliferation and apoptosis [30]. There are 18 α and 8 β subunits, each of which can bind to several partners giving rise to at least 24 distinct integrin heterodimers with different functions and ligand binding activities. Laminins are ligands for both α6β1 and α3β1 integrins. These integrins were specifically up-regulated in *Pten* null tumors. Interestingly, laminin 511 modulates human embryonic stem cell aggregation and adherence through binding of the α6β1 integrin receptor highly expressed in the membranes of undifferentiated stem cells [31], [32]. It was also reported that oval cells express integrin α6 [33]. Similarly, laminin 332 and integrin α3 were co-up-regulated in *Pten* null tumors. It was reported that cells lacking integrin α3 do not proliferate in response to laminin 332 treatment [34]. Altogether, these results suggest that laminin 332/integrin α3–induced HCC growth and that laminin 511-integrin α6β1 interaction is specific to *Pten* null tumors.

The identified HCC–associated ECM and integrin components could play an important role in the promotion of the early steps of hepatocarcinogenesis, providing a foundation for novel strategies to prevent, diagnose and treat HCC. Inhibiting the expression of ECM components or blocking their interactions with signaling integrins could serve as a means for establishing a non-permissive microenvironment that may prevent tumor development. Integrin inhibitors such as humanized antibodies or blocking peptides against integrin α5β1 are currently under clinical investigation. Our results suggest that these novel drugs should be evaluated for the treatment of HCC. In addition, integrin α3β1-laminin 332 and integrin α6β1-laminin 511 networks may be promising targets to prevent laminin-tumor cell interaction in HCC with dysregulated PTEN function.

## Methods

### Mouse Samples

The *PDGFC* Tg mice used for this study were previously described [4]. Liver tissue samples were collected by necropsy from 1.5-month old *PDGFC* Tg mice with hepatic fibrosis, 8-month old *PDGFC* Tg mice with small HCCs and from 1.5-month and 8-month old wild-type controls. Mice carrying *Pten* conditional knockout alleles were crossed with an *Albumin (Alb)-Cre*-transgenic mouse. The *Alb-Cre*-transgenic mice were genotyped using *Cre* specific primers. For this model, control animals are *Pten^loxP/loxP^*; *Alb-Cre^−^*. Liver tissue samples were collected by necropsy from 6-month old *Pten* null mice with steatosis, 9-month old *Pten* null mice with small HCCs and from 6-month and 9-month old control mice. All tissues were immediately snap-frozen in liquid nitrogen or fixed in 10% neutral buffered formalin overnight, processed to paraffin blocks, sectioned, and stained with hematoxylin/eosin or Masson's trichrome by using standard techniques. This study was carried out in strict accordance with the regulations of the U.S. National Institutes of Health. All of the work with animals was performed in adherence to the “Guide for the Care and Use of Laboratory Animals” published by the U.S. National Research Council, including the use of appropriate anesthesia whenever recommended by these guidelines. The protocol was approved and reviewed annually by the Institutional Animal Care and Use Committee of the Fred Hutchinson Cancer Research Center (File #1662). Every effort was made to minimize the number of animals required for the study and to minimize the pain and discomfort experienced.

### Protein Extraction and Separation

Liver tissues from three or four mice in each study group were separately ground on dry ice and subsequently pooled. Proteins were extracted twice from 40 mg of each pooled group in 1 ml lysis buffer (5 M urea, 2 M thiourea, 2% w/v n-Octyl-β-D-Glucopyranoside, 40 mM Tris and 1 mM phenylmethylsulfonyl fluoride). Following centrifugation at 16,100×g at 4°C for 1 hr, the pellet fraction was solubilized in Laemmli buffer and the proteins from the supernatant were separated using the Alliance 2-D Bioseparations System (Waters Corporation, Milford, MA) as previously described [13]. Briefly, an anion exchange column, BioSuite Q 10 µm, (Waters Corporation, Milford, MA) was used for the first dimension. Eight stepwise gradients were performed consisting of 0, 100 mM, 200 mM, 350 mM, 500 mM, 650 mM, 800 mM and 1000 mM NaCl. The reversed phase columns, Symmetry300 C4 3.5 µm, (Waters Corporation, Milford, MA) were used for separation of the fractions obtained from the first dimension steps. Two reversed phase columns were switched through the column selector. A total of ∼260 fractions was collected for each pooled group. Some adjacent fractions were combined leading to a final number of 34 samples for each pooled group. All fractions were lyophilized and resuspended in Laemmli buffer. Proteins obtained from the 2-D HPLC separations were further separated by 12% SDS PAGE. Gel pieces were combined into 37 individual samples for each pooled group according to protein size and abundance, dehydrated with 100% acetonitrile and dried using a speed vacuum. Gel pieces were incubated with 10 µl of 6.7 ng/µl trypsin in digestion buffer overnight at 37°C. The reaction was stopped with 15 µl of extraction buffer (2% formic acid/3% acetonitrile) and the supernatants were collected.

### Mass Spectrometry

The generated peptide samples were desalted using Symmetry C_18_ de-salting columns (Waters Corporation, Milford, MA) and subjected in duplicate to nanoflow LC-MS/MS analysis with a nano-UPLC system (Waters Corporation, Milford, MA) coupled to a hybrid 7-Tesla linear ion-trap Fourier-transform ion cyclotron resonance mass spectrometer (LTQ-FT, Thermo Scientific, Waltham, MA). Peptides were separated on a reversed phase column (75 µm×250 mm) packed with Magic C_18_AQ (5-µm 100 Å resin; Michrom Bioresources, Auburn, CA) and directly mounted on the electrospray ion source. We used a 60 min gradient from 10% to 40% acetonitrile in 0.1% formic acid at a flow rate of 300 nl/min. A spray voltage of 1600 V was applied. The LTQ-FT instrument was operated in the data-dependent mode, switching automatically between MS survey scans in the FTICR (target value 1,600,000, resolution 100,000, and injection time 1.5 s) with MS/MS spectra acquisition in the linear ion trap. The five most intense ions from the FT full scan were selected for fragmentation in the linear ion trap by collision-induced dissociation with a normalized collision energy of 30% at a target value of 10,000 (injection time 400 ms). Selected ions were dynamically excluded for 60 s. The absolute average mass accuracy for the parent ion was <5 ppm.

### Proteomic Data Analysis

Acquired data were processed using the X!Tandem search algorithm [35] and PeptideProphet and ProteinProphet statistical tools [36], [37]. The tandem mass spectra were searched against the mouse International Protein Index protein sequence database (IPI, version 3.34, http://www.ebi.ac.uk/IPI/). The following search criteria were used in all cases: trypsin specificity, 2.5 Da of mass accuracy for the parent ion and methionine oxidation as a variable modification and when specified, lysine oxidation was added as a variable modification. Relative abundance scores were calculated for individual proteins based on total peptide counts normalized to account for the total amount of protein in the mixture.

### Quantitative PCR

Total RNA was extracted from individual liver tissue samples and purified using the Trizol reagent (Invitrogen, Carlsbad, CA). RNA samples were then submitted to DNAse digestion, reverse transcription using random hexamers and real-time PCR using specific primers listed in Table S2. For each sample, the cDNA equivalent to 1 ug total RNA per 20 µl reaction was amplified with the iCycler MyiQ using SYBR Green Supermix and analyzed by MyiQ software (Bio-Rad Laboratories, Hercules, CA) and relative quantification of RNA expression was calculated with the 2^−ΔΔCt^ method. Actin quantification was used for normalization. The specificity of qPCR products was confirmed by melting curve analysis and gel-based analysis of the PCR products.

### Western Blotting

Proteins from individual liver tissues were extracted from 100 mg of liver, in 1 ml lysis buffer consisting of 5 M urea, 2 M thiourea, 2% w/v n-Octyl-β-D-Glucopyranoside (Sigma-Aldrich, St Louis, MO), 50 mM Tris (Fisher Scientific) and 1 mM phenylmethylsulfonyl fluoride (GE Healthcare, Little Chalfont, UK). The homogenate was centrifuged at 19,000× g at 4°C for 1 hour and supernatant was collected. Proteins (40 ug) were loaded onto 12% SDS PAGE gels and immunoblotting was performed using rat monoclonal anti-Nidogen 1 antibody (Santa Cruz Biotechnology, Santa Cruz, CA) at 1/100 dilution. Immunoreactivity was revealed by enhanced chemiluminescence using ECL kit (GE Healthcare, Little Chalfont, UK) and quantification was performed using ImageJ (http://rsbweb.nih.gov/ij/).

## Supporting Information

Table S1 Collagen proteins, non-collagenous ECM proteins and ECM receptors identified in *PDGFC* Tg and *Pten* null liver. The table includes for each protein: the protein and gene names, IPI and SwissProt accession numbers, the ProteinProphet score, the number and sequence of the unique peptides assigned to the protein.
(DOC)

Table S2 Primer sequences used for quantitative PCR.
(DOC)
