# Supplementary material for: Extracellular Matrix Dynamics in Hepatocarcinogenesis: a Comparative Proteomics Study of PDGFC Transgenic and Pten Null Mouse Models
Source: PLoS Genet. 2011 Jun 23;7(6):e1002147. doi: 10.1371/journal.pgen.1002147 (PMC3121762; doi:10.1371/journal.pgen.1002147)
Supplement: Table S1 — Collagen proteins, non-collagenous ECM proteins and ECM receptors identified in PDGFC Tg and Pten null liver. The table includes for each protein: the protein and gene names, IPI and SwissProt accession numbers, the ProteinProphet score, the number and sequence of the unique peptides assigned to the protein. (DOC) [file pgen.1002147.s001.doc]

**Table S1:** Collagen proteins, non-collagenous ECM proteins and ECM receptors identified in *PDGFC* Tg and *Pten* null liver. The table includes for each protein: the protein and gene names, IPI and SwissProt accession numbers, the ProteinProphet score, the number and sequence of the unique peptides assigned to the protein.

|  |  |  |  |  |  |  |
| --- | --- | --- | --- | --- | --- | --- |
| **Collagens** |  |  |  |  |  |  |
|  |  |  |  |  |  |  |
| **Protein name** | **Gene name** | **IPI #** | **Swissprot #** | **Protein Prophet** | **# unique peptides** | **Peptide sequences** |
| Collagen alpha-1(I) chain | *Col1a1* | IPI00329872 | P11087 | 1 | 29 | DGEAGAQGAPGPAGPAGER, DGLNGLPGPIGPPGPR, DRDLEVDTTLK, GDKGETGEQGDR, GDPGPQGPR, GDRGETGPAGPAGPIGPAGAR, GDRGETGPAGPPGAPGAPGAPGPVGPAGK, GDTGAKGEPGATGVQGPPGPAGEEGK, GDTGAPGAPGSQGAPGLQGMPGER, GEAGPPGPAGFAGPPGAPGAPGAPGPVGPAGK, GEPGATGVQGPPGPAGEEGKR, GEPGPPGPAGAAGPAGNPGADGQPGAK, GEPGSPGENGAPGQMGPR, GETGPAGPAGPIGPAGAR, GFSGLDGAK, GFSGLDGAKGDAGPAGPK, GLTGPIGPPGPAGAPGDK, GLTGSPGSPGPDGK, GPAGPQGPR, GPAGPQGPRGDKGETGEQGDR, GPSGERGAPGPAGPK, GPSGPQGPSGPPGPK, GQAGVMGFPGPK, GSEGPQGVR, NGDRGETGPAGPAGPIGPAGAR, NWYISPNPK, QMSYGYDEK, SAGVSVPGPMGPSGPR, SLSQQIENIR |
| Collagen alpha-2(I) chain | *Col1a2* | IPI00222188 | Q01149 | 1 | 30 | GEAGAAGPSGPAGPR, GEAGNIGFPGPK, GEAGNIGFPGPKGPSGDPGKPGER, GEPGPAGSVGPVGAVGPR, GESGNKGEPGSVGAQGPPGPSGEEGK, GESGNKGEPGSVGAQGPPGPSGEEGKR, GETGLRGDTGNTGR, GEVGLPGAPGFPGVHGEK, GEVGPAGPNGFAGPAGAAGQPGAK, GEVGPAGPNGFAGPAGAAGQPGAKGEK, GEVGPAGPNGFAGPAGAAGQPGAKGEKGTK, GLVGEPGPAGSK, GPAGPSGPVGK, GPAGPSGPVGKDGR, GPKGENGIVGPTGSVGAAGPSGPNGPPGPVGSR, GPSGPQGIR, GPSGPQGIRGDKGEPGDK, GPSGPQGIRGDKGEPGDKGHR, GSTGPAGIR, GTPGESGAAGPSGPIGSR, GVSSGPGPMGLMGPR, GVVGPQGAR, GYPGSIGPTGAAGAPGPHGSVGPAGK, LDEETGSLNK, NSIAYLDEETGSLNK, QYSDKGVSSGPGPMGLMGPR, SGQPGPVGPAGVR, SLNNQIETLLTPEGSR, TGETGASGPPGFVGEK, VGAPGPAGAR |
| Collagen alpha-1(II) chain | *Col2a1* | IPI00471183 | P28481 | 1 | 8 | EGSPGADGPPGR, GEAGAQGPMGPSGPAGAR, GQAGEPGIAGFK, VGPSGAPGEDGRPGPPGPQGAR, GAQGPPGATGFPGAAGR, GETGPAGPQGAPGPAGEEGKR, GQPGVMGFPGPK, QGDQGIPGEAGAPGLVG |
| Collagen alpha-1(III) chain | *Col3a1* | IPI00129571 | P08121 | 0.99 | 11 | GEKGEPGGAGADGVPGK, PGPLGIAGLTGAR, DGSPGGKGDRGENGSPGAPGAPGHPGPPGPVGPSGK, GEGGPPGPAGPTGSSGPAGPPGPQGVK, GEGGPPGPAGPTGSSGPAGPPGPQGVKGER, GEHGPPGPAGFPGAPGQNGEPGAK, GEKGEPGGAGADGVPGKDGPR, GPAGPNGIPGEK, GPPGTAGIPGAR, GPSGFRGPAGPNGIPGEK, GPVGPHGPPGK |
| Collagen alpha-1(IV) chain | *Col4a1* | IPI00109588 | P02463 | 1 | 11 | KPTPSTLKAGELR, AAAKGDCGGSGCGKCDCHGVK, FSTMPFLFCNINNVCNFASR, GDVGLPGMPGSMEHVDMGSMK, GEAGLPGTPGPTGPAGQK, GEQGFMGPPGPQGQPGLPGTPGHPVEGPK, GQAGFPGGPGSPGLPGPK, GSVGGMGLPGSPGEKGVPGIPGSQGVPGSPGEK, ILYHGYSLLYVQGNER, NDYSYWLSTPEPMPMSMAPISGDNIRPFISR, GDPGLSGTPGSPGLPGPK |
| Collagen alpha-2(IV) chain | *Col4a2* | IPI00338452 | P08122 | 1 | 11 | GDAGPQGPSGSGGFPGLPGPQGPK, FSTMPFLYCNPGDVCYYASR, GAPGVAGAPGPK, GEAGFFGVPGLK, GEKGDEGPMGLKGYLGLK, GISGLHGLPGTK, IAVQPGTLGPQGR, LWSGYSLLYFEGQEK, SVSIGYLLVK, GLDGFQGPSGPR, GSPGMDGFQGMLGLK |
| Collagen alpha-3(IV) chain | *Col4a3* | IPI00137938 | Q9QZS0 | 0.99 | 4 | GSPGLPGLTGPK, GRKGTSGLPGLAGRPGLTGIHGPQGDK, ALEPYISR, GPQGPSGPPGVPGSPGLSRPGLR |
| Collagen alpha-4(IV) chain | *Col4a4* | IPI00626353 | Q9QZR9 | 1 | 7 | GLPGPGCKGEPGPDGRR, GEPGDAGPPGDGGFSGER, GIQGAAGEPGLFGFLGPK, GLPGPPGLPGAPGEK, GPMGLPGLPGPPGLPGAPGEK, GFPGIPGSPGHSCER, SYWLSSAAPLPMMPLSEEEIR |
| Collagen alpha-5(IV) chain | *Col4a5* | IPI00113477 | Q63ZW6 | 1 | 3 | GDPGPPGFDVPGLPGER, GLSGQPGSPGLPGPK, GQSIQPFISR |
| Collagen alpha-6(IV) chain | *Col4a6* | IPI00113475 | Q9ESQ1 | 0.98 | 3 | GLNGKPGMLGPK, GTSGFPGAPGLPGISGHPGK, GSSGPVGFPGLPGLPGLPGADGLK |
| Collagen alpha-1(V) chain | *Col5a1* | IPI00128689 | O88207 | 1 | 10 | TGPIGPQGAPGKPGPDGLR, GEKGEAGPSGAAGPPGPKGPPGDDGPK, GNSGGDGPAGPPGER, GPAGPMGLTGR, ALVDGCATK, ANQDTIFEGIGGPR, FGGGGDAGSKGPMVSAQESQAQAILQQAR, GPNGPQGPTGFPGPK, GTPGKPGPR, TGPIGPQGAPGKPGPDGLR |
| Collagen alpha-2(V) chain | *Col5a2* | IPI00121120 | Q3U962 | 1 | 5 | GLTGNPGVQGPEGK, GPAGPAGEPGK, GSSGDLGKPGEAGNAGVPGQR, GAHGMPGKPGPMGPLGIPGSSGFPGNPGMK, LGPQGAPGK |
| Collagen alpha-3(V) chain | *Col5a3* | IPI00626350 | Q9JLI2 | 0.97 | 2 | RGSPGRMGPEGR, GLPGDTGPK |
| Collagen alpha-1(VI) chain | *Col6a1* | IPI00339885 | Q04857 | 1 | 17 | DAEEVISQTIDTIVDMIK, EGPVGIPGDSGEAGPIGPK, FEPGQSHAGVVQYSHNQMQEHVDMR, GLEELLIGGSHLK, GLEELLIGGSHLKENK, GVLYQTVSR, IALVITDGR, LSIIATDHTYR, LSKDELVK, NLVWNAGALHYSDEVEIIR, RNFTAADWGHSR, SDGNSQGATAEAIEK, TAEYDVAFGER, VAVVQYSGQGQQQPGR, VFSVAITPDHLEPR, VLLFSDGNSQGATAEAIEK, VPNYQALLR |
| Collagen alpha-2(VI) chain | *Col6a2* | IPI00621027 | Q02788 | 1 | 24 | ATYLNSFSHVGTGIVHAINNVVR, DDDLNLR, DIANSPHELYR, EKDFDSLAQPSFFDR, ETCGCCDCEK, FAYNQLIK, FVEDVSR, GEPGPPGPEGGR, GPQGALGEPGK, GSKGYQGNNGAPGSPGVK, KQNVVPTVVAVGGDVDMDVLTK, LDDERVNSLSSFK, LFAVAPNR, LGEQNFHK, NFVINVVNR, NLEWIAGGTWTPSALK, NLNEQGLRDIANSPHELYR, NNYATMRPDSTEIDQDTINR, QDWMQLFIDTFK, RDDDPLNAR, RFVEDVSR, VFAVVITDGR, VGVVQYSHEGTFEAIR, VNSLSSFK |
| Collagen alpha-3(VI) chain | *Col6a3* | IPI00830749 | O88493 | 1 | 67 | EVQVSEVTENSAR, ALEFVAR, ALNLGYALDYALR, DFVTNLVNSLDVGSDNIR, DQNVFVSQK, DSFQEVLR, DVVFLIDGSR, EELSSLKPILTPSTGAGVGSK, ELGTIQQVISER, EVYSFASEPNDVFFK, FASEIVDTVYEDGDSIR, GETGDDGRDGVGSEGR, GETGDDGRDGVGSEGRR, GGGGTAGAPGERGRTGPLGR, GGPGQPGFEGEQGTR, GGSQVYIGNALEYVLK, GVVPFIFQAK, IASNSATAFR, IEDGVPQHLVLFLGGK, IEDNVQQFLVLLVAGR, IEEGVPQFLVLISSGK, IGDLQSQIVSLLK, IGVVQFSNDVFPEFYLK, IIEELDVKPDGTR, IMVLMLTGDMQR, ISLSPEYVYSVSTFR, IVEYLDIGFDTTR, LDERVPQIAFVITGGK, LLPSFVSSENAFYLPPDLR, LLTPITTLTSQQIHQILASTR, LLVLITGGK, LMHLEFGR, LNLLDLDYELAEQLDNIAEK, LSDAGITPLFLTSQEDR, LTLLGGPTPNTGAALEFVLR, NAGPEFQYIR, NANPSELEQIVLSPAFILAAESLPK, NHFVPEAGSR, NIDSEEVGK, NNLFTSSAGHR, PSELEQIVLSPAFILAAESLPK, QAGAQALILVGLER, QFGVAPLTIAR, QQHGVMEVNKR, QQSLETAMSFVAR, RDLTDFLK, RMTQLQGTMGLGNAIEYTIENIFESAPNPR, SALLDSIQNLQVALTSK, SDDEVDDSAVELK, SFGDLQEQILPYLVGVAQR, SQRPYVLTADTLK, SQSSVLEAIR, SVEDAQDVSLALTQK, TDLQTITNDPR, TLTGTTEESKR, VAIAQFSDDVR, VALVQYSDR, VAVFFSNKPTR, VAVVTYNNEVTTEIR, VDIILNR, VGIEHLLR, VGLVQFSDTPVTEFSLDTYQTK, VGLVQYNSDPTDEFFLR, VPQIAFVITGGK, VVESLDVGPDR, VVIHFTDGADGDMADLYR, YPPSVVESDAADIVFLIDSSDAVKPDGIAHIR |
| Collagen alpha-1(VII) chain | *Col7a1* | IPI00134652 | Q63870 | 1 | 4 | DPEAPLVVPGLR, GEPGRPGDPAVGPGGAGAKGEK, GQGVKLFAVGIK, VAIEEPGPGLAR |
| Collagen alpha-1(VIII) chain | *Col8a1* | IPI00399464 | Q00780 | 1 | 1 | GPIGAPGMGGPP |
| Collagen alpha-1(X) chain | *Col10a1* | IPI00109296 | Q05306 | 0.99 | 2 | GDIGPAGLPGPR, GIPGNHGIPGAK |
| Collagen alpha-1(XIV) chain | *Col14a1* | IPI00330632 | Q80X19 | 1 | 25 | TNQMNLQNTATK, NLVVDDETATSLR, NFLENLVTAFNVGSEK, YTAILNQIPSQSSSIR, LQELEGPSVSIMQK, GDLQSQAMVR, ESGVELFAIGVK, NLMSSTEYQIAVFAVSAHTASEGLR, VVYYPTR, GGKPEEVVVDGSVSSTVLK, HVFFVDDFDAFK, IVLESLQDTQAQESTVGGGVNR, VTVTPVYTVGEGVSVSAPGK, IINFLYSTVGALDK, LMWIPVYGGK, IGILITDGK, YLHPEGLPSDYTMSFLFR, ISNVGSNSAR, IEWHLNAFNTKDEVIDAVR, GGNTLTGLALNFIFENSFKPEAGSR, IGLAQYSGDPR, ASALATIGPPTELITSEVTAR, TEFKLDSYK, TLPSSGPQNLR, ILPDTPQEPFALWEILNK |
| Collagen alpha-1(XV) chain | *Col15a1* | IPI00409035 | O35206 | 1 | 7 | GADGTSTMGPPGPR, FTGSIQQLTIYSDPR, EAAVFSVPVMTNR, TTDMAVTGFASPLSTGK, AFLSSHLQDLSTVVR, AAGLLSTFR, FGLPIVNLK |
| Collagen alpha-1(XVI) chain | *Col16a1* | IPI00648306 | Q8BLX7 | 1 | 6 | GEPGSPGFGLPGK, LMLSVAGR, SGMPGGPGKSGSMGPIGPPGPAGER, GSPGPPGPIGPPGFPGAVGSPGLPVR, GEPGSPGFGLPGKQGKAGER, EGPGGKPGKPGVPGTK, |
| Collagen alpha-1(XVIII) chain | *Col18a1* | IPI00131476 | P39061 | 1 | 14 | TETTGATGQASSLLSGR, ASQGLELER, GSVPIVNLKDEVLSPSWDSLFSGSQGQLQPGAR, ILNVAQGIR, GAGLFVGQAGTADPDKFQGMISELK, GSVPIVNLK, GTGNEVAALQPPLVQLHEGSPYTR, DGNSLSPLNPLVWLWPPK, FGINGSYAPGPAGLPGVPGK, LQDLYSIVR, QTVSVPGPPGPPGPPGPPGAMGASAGQVR, DEVLSPSWDSLFSGSQGQLQPGA, IFSFDGR, AVGLSGTFR |
| Putative protein COL22A1 | *Col22a1* | IPI00757330 | D3Z3P0 | 1 | 5 | GEKGTAGEEGSPGPAGPR, GEVGLPGAPGFPGVHGEK, GTAGEEGSPGPAGPR, GTPGIPGSPGSR, GPAGPQGPR |
| Collagen alpha-1(XXVII) chain | *Col27a1* | IPI00408491 | Q5QNQ9 | 1 | 6 | RGNPGVAGLPGAQGPPGFK, GNMGLPGLSGN, KPEPLSPGK, DSMLDPQGSFLLGK, KPEPLSPGK, TPSPSSSASLANSTR |
| Collagen alpha-1(XXVIII) chain | *Col28a1* | IPI00357842 | Q2UY11 | 0.99 | 5 | GEVGQMGPTGPR, GPEGMPGKGQPGPK, GLPGEGFPGPK, LIIEICGCGPK, MGAPGPIGIGEPGQPGPR |
|  |  |  |  |  |  |  |
|  |  |  |  |  |  |  |
| **Non-collagenous ECM**  **proteins** | |  |  |  |  |  |
|  |  |  |  |  |  |  |
| **Protein name** | **Gene name** | **IPI #** | **Swissprot #** | **Protein Prophet** | **# unique peptides** | **Peptide sequences** |
|  |  |  |  |  |  |  |
| Laminin subunit alpha-5 | *Lama5* | IPI00116913 | Q61001 | 1 | 10 | AVEASNAYSSILQAVQAAEDAAGQALR, DLGAQGAVAEAELAEAQR, FGFNPLEFENFSWR, FMNQEVETQR, GQLQLVEGNFR, GTQDNNLLYYR, MGQGSPGDALVPSGEQLR, QATGDYMGVSLR, TTPMLQLQPEEPSR, VVLEVASEAGR |
| Laminin subunit beta-1 | *Lamb1-1* | IPI00338785 | P02469 | 1 | 2 | VETLSQVEVILQQSAADIAR, CVCNYLGTVK |
| Laminin subunit beta-2 | *Lamb2* | IPI00119065 | Q61292 | 1 | 14 | AGNSLAASTAEETAGSAQSR, ALVEGGGILSR, AMDYDLLLR, CGGLGCSGAAAPADLALGR, DGFFGLSASDPR, GQVEQANQELR, HSNFLGAYDSIR, IQNVVTSFAPQR, LGMVQAIMSAR, LQELEGTYEENER, LREGQEVEFLVTSLPR, VLDISIPASPEQIQR, VVQDLAAR, YSEIEPSTEGEVIYR |
| Laminin subunit beta-3 | *Lamb3* | IPI00788345 | Q61087 | 1 | 2 | CDCNGHSETCHFDPAVFAASQGTNGGVCDNCR, VENVASSSGPMR |
| Laminin subunit gamma-1 | *Lamc1* | IPI00400016 | P02468 | 1 | 6 | EAQLALGNAAADATEAK, LSAEDLVLEGAGLR, NTIEETGILAER, SRVESTEQLIEIASR, TAAEEALRR, VSVPLIAQGNSYPSETTVK |
| Laminin subunit gamma-2 | *Lamc2* | IPI00117115 | Q13753 | 0.98 | 3 | ACNCSPMGSEPGECR, APLMAPGKTLPCGITK, CIYNTAGVYCDQCK |
| Decorin | *Dcn* | IPI00123196 | P28654 | 1 | 6 | ASYSAVSLYGNPVR, DLHTLILVNNK, KASYSAVSLYGNPVR, NSGIENGAFQGLK, VPAGLAQHK, YWEIFPNTFR |
| Nidogen-1 | *Nid1* | IPI00111793 | P10493 | 1 | 4 | EDLSPFIIQMAAEYVQR, TNSVIAMDLAISK, VLEGLQYPFAVTSYGK, VLFDTGLVNPR |
| Perlecan | *Hspg2* | IPI00515360 | Q05793 | 1 | 32 | AEMLQALASLEAVLLQTVYNTK, HPTPLALGQFHTVTLLR, GHTPTHPGTLNQR, IEGNTLVIPR, AMLQVHGGSGPR, FDAGSGMATIR, QPDFISFGLVGGRPEFR, SPAYTLVWTR, VIPYFTQTPYSFLPLPTIK, QLISTHFAPGDFQGFALVNPQR, AASISAVSLEVAQPGPSSGPR, NSQLTGGFTVEPVHDGAR, VGGHLRPGIVQSGSIIR, AMDFNGILTIR, TSTADGLLLWQGVVR, YELGSGLAVLR, RFLVHDAFWALPK, GMVFGIPDGVLELVPQR, GMLEPVQKPDVILVGAGYR, SIEYSPQLEDASAK, IQVVVLSASGANSVPVR, SVVPQGGPHSLR, GGSLPPHAQVHGSR, ALSSAGQHVAR, IAHVELADAGQYR, VSGISMDVAVPENTGQDSAR, LSFDQPNDFK, FLVHDAFWALPK, DPSPGQPSNFIVPFQEQAWQRPDGQPATR, VDSYGGFLR, ESLEVQIHPSR, AFAYLQVPER |
|  |  |  |  |  |  |  |
|  |  |  |  |  |  |  |
| **ECM receptors** |  |  |  |  |  |  |
|  |  |  |  |  |  |  |
| **Protein name** | **Gene name** | **IPI #** | **Swissprot #** | **Protein Prophet** | **# unique peptides** | **Peptide sequences** |
|  |  |  |  |  |  |  |
| CD44 antigen | *CD44* | IPI00266155 | P15379 | 1 | 3 | YGFIEGNVVIPR, YAGVFHVEK, NQDVMGVSGGGC |
| Integrin alpha-IIb | *Itga2b* | IPI00315155 | Q9QUM0 | 0.95 | 1 | GPQALSTPTLLLTGTQLYGR |
| Integrin alpha-3 | *Itga3* | IPI00468674 | Q62470 | 1 | 2 | LGLPGLATFGYSLSGK, AAFLSEQLQPLSR |
| Integrin alpha-5 | *Itga5* | IPI00115976 | P11688 | 1 | 11 | TPDFFGSALR, AGTSLWGGLR, ILESSLYSAK, VTAPLEAEYSGLVR, DGVSVLVGAPK, SLQWFGATVR, DLDGNGYPDLIVGSFGVDK, QATLTQTLLIQNGAR, LQVATAVQWTK, AQLKPPATSDA, MPYQILPR |
| Integrin alpha-6 | *Itga6* | IPI00331413 | Q61739 | 0, 99 | 2 | LIATFPDTLTYSAYR, LNYLDILLR |
| Integrin alpha-8 | *Itga8* | IPI00345112 | A2ARA8 | 1 | 3 | SNQWFGATVR, VLIYNGNPR, GDADIDKNDYPDLLVGAFGK |
| Integrin beta-1 | *Itgb1* | IPI00132474 | P09055 | 1 | 4 | SAVTTVVNPK, CTDPKFQGPTCETCQTCLGVCAEHK, LSENNIQTIFAVTEEFQPVYK, LRPEDITQIQPQQLLLK |
| Integrin beta-5 | *Itgb5* | IPI00229516 | O70309 | 1 | 2 | CPTCPDACSSK, CVCGQCQCTEPGAFGETCEK |
