# Supplementary material for: Extracellular Matrix Dynamics in Hepatocarcinogenesis: a Comparative Proteomics Study of PDGFC Transgenic and Pten Null Mouse Models
Source: PLoS Genet. 2011 Jun 23;7(6):e1002147. doi: 10.1371/journal.pgen.1002147 (PMC3121762; doi:10.1371/journal.pgen.1002147)
Supplement: Table S2 — Primer sequences used for quantitative PCR. (DOC) [file pgen.1002147.s002.doc]

**Table S2:** Primer sequences used for quantitative PCR.

| **primers** | **sequences** | **size** |
| --- | --- | --- |
| collagen 4a2 F | g a g t c g t a g g a c a g a a a g g | 261bp |
| collagen 4a2 R | c t c t c a t t g a a t c t t c a t c t c |  |
|  |  |  |
| collagen 15a1 F | c a g a a c t t g g a t t t g a g a t t | 220bp |
| collagen 15a1 R | g t g a t g t t g a t c a a a g a a c t g |  |
|  |  |  |
| collagen 6a2 (canonical) F | t a c c t c a a t t c c t t t t c t c a | 239bp |
| collagen 6a2 (canonical) R | c t g a t c t t a g t a a g c a c a t c c |  |
|  |  |  |
| collagen 6a2 (splice variant) F | a g a t c g t g t g t c c a g a a c t | 278bp |
| collagen 6a2 (splice variant) R | a c c a g t t t a a a g g t g t c a a t |  |
|  |  |  |
| collagen 18a1 (canonical - NC1-764) F | g t t t a c t c t c t g t t c c t t c c t | 267bp |
| collagen 18a1 (canonical - NC1-764) R | c t a c c t c c a c a c t g t c t g t a t |  |
|  |  |  |
| collagen 18a1 (variant - NC1-301) 0616 F | c t c a c c a g t t t g g t c t t g | 120bp |
| collagen 18a1 (variant - NC1-301) 0616 R | t t g t g a g a t c t t c t c a g g t a g |  |
|  |  |  |
| nidogen 1 F | a t a t t g a t g a g t g t t c a g a g c | 169bp |
| nidogen 1 R | a g a c c a g t t t c a c a g t a g t t g |  |
|  |  |  |
| laminin alpha-5 F | a t g c t t a c a g c t c c a g t t a c | 153bp |
| laminin alpha-5 R | t g t t g t a g a a g a g a g a c a a g g |  |
|  |  |  |
| Integrin alpha-6 F | c a g c a a c c t t g a a t a t a c a g t | 160bp |
| Integrin alpha-6 R | t t c t t t c t t g a g t t g t g a g a c |  |
|  |  |  |
| Integrin alpha-8 F | c g g a t a t a c t g t t g t g g t a t c | 110bp |
| Integrin alpha-8 F | c c a c t t c t c t a g g g t t a c t t t |  |
|  |  |  |
| Pdgfa F | g a c g g t c a t t t a c g a g a t a c | 185bp |
| Pdgfa R | t c t t c c t g a c a t a c t c c a c t |  |
|  |  |  |
| Pdgfc F | g c a t g a g a g a g t t g t c a c t a t | 182bp |
| Pdgfc R | a c t t g c a t a t a t c g t c t t c t g |  |
